# Vietnam: Neglected tropical diseases in an emerging and accelerating economy

**DOI:** 10.1371/journal.pntd.0010140

**Published:** 2022-02-17

**Authors:** Kala Pham, Peter J Hotez

**Affiliations:** 1 Departments of Biology and Biochemistry, University of Houston, Houston, Texas, United States of America; 2 Houston Premedical Academy, University of Houston and Baylor College of Medicine, Houston, Texas, United States of America; 3 Departments of Pediatrics and Molecular Virology and Microbiology, National School of Tropical Medicine, Baylor College of Medicine, Houston, Texas, United States of America; 4 Department of Biology, Baylor University, Waco, Texas, United States of America; 5 Hagler Institute of Advanced Study, Texas A&M University, College Station, Texas, United States of America; 6 James A Baker III Institute of Public Policy, Rice University, Houston, Texas, United States of America; Walter and Eliza Hall Institute of Medical Research, AUSTRALIA

The Socialist Republic of Vietnam (Vietnam) has made tremendous strides in reducing its disease burden from tropical infections, including malaria and neglected tropical diseases (NTDs). It now joins South Korea, Japan, and the eastern half of China as nations or regions that have achieved great successes in disease control through a combination of economic reforms, mass drug preventive treatment programs, and other public health interventions [1–3]. Here, we provide a brief update on these activities, including Vietnam’s prospects for disease elimination or, in some instances, disease reemergence.

## Doi Moi

**Fig 1:**
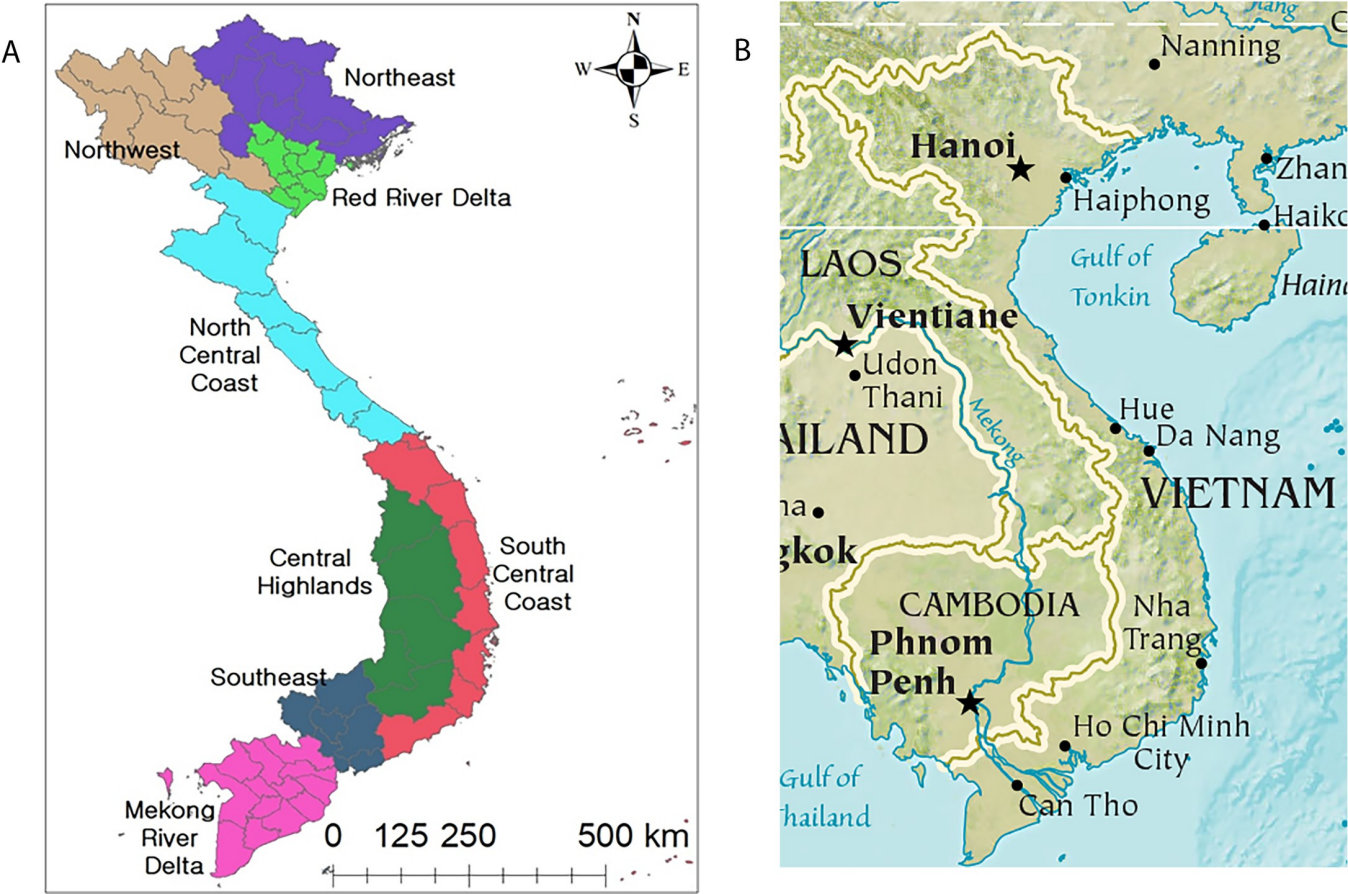
The geographic regions of Vietnam. **(A)** Named geographic areas. From Lee and colleagues: https://journals.plos.org/plosone/article?id=10.1371/journal.pone.0194943. **(B)** Topographic map showing mountainous areas. Modified from CIA Factbook: https://www.cia.gov/the-world-factbook/maps/world-regional/; https://www.cia.gov/the-world-factbook/static/35ffc797934a80da7d944dfa89624581/southeast_asia_phy.pdf.

Beginning in the middle 1980s, the government of Vietnam implemented a series of economic reforms under the banner of Doi Moi (“restoration”), which included market liberalizations and encouragement of private investments both domestically and from overseas. Prior to this period, Vietnam was considered among the poorest countries in Asia, but over the last 20 years, its economy has almost tripled, as its population has grown by approximately 25% to almost 100 million people [4]. More than 45 million people in Vietnam have escaped poverty over this period [4]. However, much of this economic growth has occurred disproportionately in urban areas, leaving approximately 6% of the population remaining in extreme rural poverty. Among these approximately 6 million people, 86% are considered ethnic minorities or indigenous groups [5]. Vietnam’s ethnic minorities who remain in poverty live predominantly in remote mountainous areas found in northern, western, and central regions of the country (Fig 1) [2].

In all, Vietnam has 54 ethnic groups, with the Kinh considered the largest [6]. While Vietnam’s ethnic minorities have protected status by the government of Vietnam, they are also recognized as socioeconomically disadvantaged, having benefited less from Doi Moi than the rest of the population [6]. They also disproportionately suffer from NTDs [7]. Compounding these vulnerabilities is Vietnam’s low level of physical capital investment compared to other Association of Southeast Asian Nations (ASEAN) nations and environmental degradation due to overfishing and deforestation [3]. Vietnam is also under threat due to climate change, with widespread flooding or even complete inundation from rising seas in its southern delta region and other low-lying coast areas [8]. Another expected consequence of climate change is warming temperatures that may promote the emergence of dengue and other vector-borne NTDs [9]. Therefore, Vietnam experiences social and physical determinants that can either promote or reduce its NTDs.

## NTDs declining

Data from the Institute of Health Metrics and Evaluation find that overall, the burden of disease (in terms of disability-adjusted life years [DALYs]) from malaria and NTDs has fallen by 62% between 2000 and 2019. This includes an almost 80% decline in deaths, mostly due to malaria-related deaths [10]. Shown in Table 1 are the declines in specific NTDs, as well as malaria.

**Table 1:** NTDs that have declined in age-adjusted prevalence or incidence*.

Disease	Age-adjusted prevalence cases or incidence in 2000	Age-adjusted prevalence or incidence in 2019	% Decline
Malaria*	275,202.78	17,600.35	93.6
LF	2,669,033.05	767,798.69	71.23
Trachoma	40,735.88	18,876.41	53.66
Soil-transmitted helminths	44,185,437.34	18,477,777.10	58.18
Rabies*	166.83	93.05	55.77

*Denotes incidence data [10–14].

LF, lymphatic filariasis; NTD, neglected tropical disease.

### Malaria

Prior to Doi Moi, malaria was highly endemic in Vietnam, but, together with economic reforms, a national malaria control program was established in 1992. Comprised of prompt case detection and treatment, indoor residual spraying, and the widespread distribution of insecticide-treated bednets, the incidence of new malaria cases has declined precipitously [6,15,16]. Partial support for Vietnam’s malaria program has been provided by the Global Fund to Fight AIDS, Tuberculosis and Malaria to the National Institute of Malaria, Parasitology, and Entomology (NIMPE) of the Ministry of Health (MOH) [17]. These funds have been especially helpful for supporting surveillance systems and monitoring malaria among migrants and other mobile populations [17]. However, malaria remains a significant public health threat in southern and central mountainous regions where ethnic minorities live, as well as migrants [6]. Approximately two-thirds of Vietnam’s malaria cases are caused by *Plasmodium falciparum*, with *Plasmodium vivax* making up the rest [18]. Of greatest concern is the documented emergence of artemisinin resistant malaria strains, especially in highland areas [15,16,19,20].

### Lymphatic filariasis

Perhaps Vietnam’s most successful NTD control program has been its National Program to Eliminate lymphatic filariasis (LF). Launched in 2001 by the NIMPE-MOH, and with technical support from the World Health Organization (WHO) and the Global Programme to Eliminate LF [21], the national program has recently achieved elimination status [22]. The major approach includes mass drug administration with diethylcarbamazine citrate and albendazole combination therapy, or triple therapy by adding ivermectin, together with improvements linked to economic gains, including housing and water drainage infrastructure [23–25]. Since 2011, these activities accelerated through support of the United States Agency for International Development (USAID) NTD Program and its FHI360 and RTI International contractors [22]. The NIMPE-MOH is partnering with the US Centers for Disease Control and Prevention (CDC) and the Atlanta-based Task Force for Global Health to conduct operational research to help sustain and monitor LF elimination [26].

### Soil-transmitted helminth infections

The 3 major soil-transmitted helminth infections—ascariasis, trichuriasis, and hookworm infection—are the most common NTDs, with more than 10% of the population of Vietnam infected. *Necator americanus* is a predominant hookworm species in Vietnam, but *Ancylostoma ceylanicum*—an often forgotten third hookworm species—is also widespread [27,28]. Vietnam’s helminth infections are concentrated in agricultural communities, particularly the Red River Delta region, due to the use of human excreta (night soil) as fertilizer and contact with contaminated water [29–31]. While the Vietnamese government has banned the use of night soil, such practice is common and low cost; therefore, it is difficult to enforce [29]. Lack of access to wastewater infrastructure, latrines that lack a chamber for long-term excreta storage, inconsistent access to commercial inorganic fertilizers, and absence of proper compost procedures all contribute to the persistence of helminth infections [29,32]. Notably, they are higher in Northern Vietnam than Southern Vietnam, either due to poverty or environmental conditions [30,33]. In response, the NIMPE-MOH provides 3 million or 5 million albendazole or mebendazole tablets annually, especially for school-age children [34,35]. However, the role of mass drug administration versus economic gains and improvements in water and sanitation and hygiene (WASH) in Vietnam’s declining prevalence is unclear. In addition, the effectiveness of drug treatments (albendazole or mebendazole) is not consistent among the 3 helminth infections [36–38]. Beyond the 3 major soil-transmitted helminth infections highlighted above, strongyloidiasis is also endemic, especially in the rural central highlands, but even in major urban areas. The highest prevalence of strongyloidiasis and evidence of clinical symptoms occurs among older rural populations [39,40].

### Trachoma

Trachoma is another example of an NTD that has been effectively reduced through collaboration between USAID and the Vietnam MOH in preventive treatments with azithromycin, together with other measures. Trachoma has decreased significantly in the past years [41–43]. An ENVISION partners collaboration with the FHI 360 END project, RTI International, Fred Hollows Foundation, and the Vietnam National Institute of Ophthalmology has provided trained eye health staff and surgery procedures for trachoma from 2011 to 2016 [44]. The Fred Hollows Foundation began working in Vietnam in 1992 and since then has developed key partnerships to perform 282,000 trichiasis surgeries [45]. Additionally, WHO SAFE strategy has improved health practices significantly, which contributed to the reduction of trachoma [46]. The SAFE strategy was initiated in 1996 as part of WHO Alliance for Global Elimination of Trachoma by 2020, which worked to help countries reduce trachoma through training staff and improving health practices and programs [47,48]. The strategy has proved effective, leading to a steady decrease in trachoma prevalence; however, reinfection can occur [49]. The construction of improved sanitation and water management/facilities has also contributed to the decline of trachoma [50].

### Rabies

Canine rabies is on the decline, but it remains endemic, with foci in Southern Vietnam, specifically the Mekong and Southeast Central Coast Region [51,52]. The MOH and Ministry of Agriculture and Rural Development have invested significant resources to control rabies, leading to its reduction [52]. Vietnam’s Prime Minister created the National Rabies Program in 1996, which created support and resources for rabies prevention and control. Since then, Vietnam has increased postexposure prophylaxis (PEP) centers across the country [52]. Support from WHO, World Organization for Animal Health (OIE), and the CDC have increased Vietnam’s capabilities to increase rabies awareness and improve dog surveillance, including a pilot prevention program in Thai Nguyen Province that incorporates dog registration and management [52,53].

## NTDs rising

Several NTDs are increasing or reemerging. These are listed in Table 2.

**Table 2:** NTDs that have plateaued or increased in age-adjusted prevalence or incidence*.

Disease	Age-adjusted prevalence cases or incidence in 2000	Prevalence or incidence in 2019	% Increase
Cysticercosis	23,012.42	32,921.94	43.06
Food-borne trematodiases	637,157.76	940,349.37	47.58
Cystic echinococcosis	10.38	12.57	21.09
Dengue*	967,575.13	1,038,967.72	7.38

* Denotes incidence data [54–57].

NTD, neglected tropical disease.

### Food-borne trematodiases

The prevalence of food-borne trematodes, especially liver fluke infection caused by *Opisthorchis viverrini* or *Clonorchis sinensis*, and intestinal fluke infections caused by *Fasciolopsis buski*, has increased [58,59]. Liver fluke is also an important cause of cholangiocarcioma. Opisthorchiasis is found predominantly in central southern Vietnam, whereas clonorchiasis is in northern Vietnam [60]. Vietnam’s aquaculture plays a dominant role in its economy, with freshwater fish aquaculture increasing exponentially [60]. However, the rapid rise in aquaculture is fueling the emergence of fluke infections [60,61]. For example, farmers who work on small-scale fish farms or nurseries often use livestock manure or night soil as fertilizer to help increase the growth of plankton, a food source for the fish [61].

Still, another factor is human behavior around consumption of traditional dishes containing inadequately cooked fish or fish pastes with condiments [61]. In a study conducted in Northern Vietnam, older individuals knew this risk and continued eating raw fish, because they knew drug treatment was available [62]. Many traditional dishes also utilize raw fish [58]. Furthermore, 25.8% of household members were found to have not eaten raw fish, but were infected due to cross-contamination via sharing food [62]. Finally, climate change leads to more frequent flooding, causing bodies of water with foodborne trematodes to contaminate other water supplies [62].

In summary, rising agriculture that still clings to ancient practices, including fishing practices which use feces for fertilization; increased consumption of fish because of increasing affluence by a population with a tradition of eating raw fish; and increased flooding from climate change have contributed to the rise of food-borne trematode infections.

### Cysticercosis (and African swine fever)

Cysticercosis has also increased. Vietnamese citizens in peri-urban and rural areas usually have free-roaming pigs [63]. Together with open defecation using outdoor latrines, the use of night soil for agriculture maintains or accelerates this infection [47]. Similar to aquaculture, husbandry makes up a large percentage of Vietnam’s gross domestic product (GDP) and produces nearly 3,800 million tons of meat products annually [64]. Two primary types of pig and cattle husbandry practices exist: commercial farming and backyard husbandry. In rural regions, backyard husbandry practices dominate [65]. Meat inspection is only carried out at slaughter points that operate at the district level and/or clusters of large villages [63]. Vietnam’s pork production for traditional and commercial markets have a poor supply chain; therefore, the weak linkages between actors and poor hygienic practices in these chains create risk [66]. Most slaughterhouse workers seldom go through food safety training [66]. Overall improved sanitation and meat inspection/control are needed to decrease cysticercosis incidence and transmission [67]. Education and training on food safety risks and proper handling among pork value chain actors are other necessary priorities [66]. Echinococcosis is another larval cestode infection, but it is considered rare in Vietnam [68]. However, sporadic cases of hydatid disease in the heart and lung from the species *Echinococcus ortleppi* have been identified [68].

### Dengue

Dengue epidemics now occur regularly [69]. Following a large-scale dengue fever outbreak in 2017, Vietnam recorded its highest number of dengue cases of 320,000 in 2019 [70,71]. The high population density in urban and suburban areas increases transmission and vector growth [72,73]. Climate change produces increasingly favorable precipitation, temperature, and humidity for dengue to spread [69,74]. Limited government control has curtailed improvements in dengue transmission [72]. However, because outbreaks of dengue are occurring in more frequent cycles, favorable conditions of weather, a dense human population, and rapid urbanization, there is an increased need for better governmental policy and education, including risk control, training healthcare workers to recognize dengue symptoms, and engaging local authorities [72,73]. Dengue and malaria are both mosquito borne, but the decrease in malaria can be attributed to drug treatments with artemisinin and effective centralized health programs. Both of these elements are missing with respect to dengue control. Beyond dengue, other arbovirus infections are present. For example, an Asian lineage of Zika virus infection has been detected in Vietnam and linked to microcephaly [75], and there is evidence for previous epidemics of chikungunya [76]. Japanese encephalitis is the major cause of viral encephalitis in Vietnam [77]. Transmitted by Culex mosquitoes, pigs are considered an important reservoir host [77].

## Concluding statement

NTDs exhibiting the greatest declines in Vietnam appear to be those illnesses vulnerable to mass drug administration. However, given the established impact of economic improvements in also promoting reductions in the NTDs (as noted in other East Asian nations), it is difficult to confirm the contribution of mass treatments and other public health interventions. Still, another unresolved issue is whether the reductions in NTDs lead to economic improvements or vice versa. It is likely these 2 aspects are mutually reinforcing. Of interest is our finding that the impact of mass drug administration on educational attainment and development is greater in middle-income countries exhibiting lower worm burdens, compared to fragile nations with excessively high worm burdens [78]. The basis of this observation is not known, but it has been suggested that there is an accelerant effect as economies begin to improve and burdens of disease from worms diminish. This possibility is consistent with the current situation in Vietnam.

By contrast, 2 NTDs linked to agriculture and animal husbandry, liver fluke infection and cysticercosis, respectively, appear to be increasing. Paradoxically, the rises in these NTDs may reflect increases in economic development and access to expanding food sources. While this promotes food security, so far public policies to ensure these expanding agricultural practices can be conducted safely and with attention to parasite control remain lagging. This situation has also been noted in parts of China and other emerging economies of Asia. Climate change appears to accelerate these trends, as it does for dengue and vector-borne diseases. The high transmissibility of arbovirus infections will increase the reliance on the development of new vaccines.

In summary, the one-two punch of economic gains and mass drug administration is producing dramatic public health benefits in Vietnam as they have in other nations achieving middle-income status. By prioritizing NTDs, Vietnam could become a leading influencer in the Southeast Asian region. But these improvements must be accompanied by public policies around food security to control commensurate rises in food-borne trematodiases and cysticercosis and for practices to ensure the reductions in vector-borne NTDs.
